# Extracellular vesicles in the treatment and diagnosis of breast cancer: a status update

**DOI:** 10.3389/fendo.2023.1202493

**Published:** 2023-07-17

**Authors:** Xiaoying Zhang, Caizheng Wang, Jiahui Yu, Jiawen Bu, Fulv Ai, Yue Wang, Jie Lin, Xudong Zhu

**Affiliations:** ^1^ Department of General Surgery, Huangyan Hospital, Wenzhou Medical University, Taizhou, Zhejiang, China; ^2^ Department of Ultrasound, Shengjing Hospital of China Medical University, Shenyang, Liaoning, China; ^3^ Department of Oncology, Shengjing Hospital of China Medical University, Shenyang, Liaoning, China; ^4^ Department of General Surgery, Cancer Hospital of China Medical University, Liaoning Cancer Hospital and Institute, Shenyang, Liaoning, China

**Keywords:** breast cancer, extracellular vesicles, proliferation, metastasis, chemoresistance, biomarkers

## Abstract

Breast cancer is one of the leading causes of cancer-related death in women. Currently, the treatment of breast cancer is limited by the lack of effectively targeted therapy and patients often suffer from higher severity, metastasis, and resistance. Extracellular vesicles (EVs) consist of lipid bilayers that encapsulate a complex cargo, including proteins, nucleic acids, and metabolites. These bioactive cargoes have been found to play crucial roles in breast cancer initiation and progression. Moreover, EV cargoes play pivotal roles in converting mammary cells to carcinogenic cells and metastatic foci by extensively inducing proliferation, angiogenesis, pre-metastatic niche formation, migration, and chemoresistance. The present update review mainly discusses EVs cargoes released from breast cancer cells and tumor-derived EVs in the breast cancer microenvironment, focusing on proliferation, metastasis, chemoresistance, and their clinical potential as effective biomarkers.

## 1 Introduction

Breast cancer is the most frequently diagnosed cancer in women and severely affects their physical and mental health. It is additionally the leading cause of cancer-related deaths in women (1). Therefore, clarifying mechanisms underlying the development of breast cancer is currently the focus of basic research (2, 3). Extracellular vesicles (EVs) are involved in intracellular communication by carrying important bioactive molecules and play a crucial role in breast cancer development (4–6). Although their nomenclature remains controversial, EVs are classified based on their size, biogenesis, or release pathway into the following: exosomes of endosomal origin, microvesicles of plasma membrane origin (referred to as small EVs [SEVs]), and apoptotic EVs (7). EVs are thought to be carriers to transfer the contents and mediate a wide range of biological responses in intercellular communication, cancer development, and cancer metastasis in the immune system. In addition, they are extensively involved in information exchange within the organism by transferring functional bio-signaling molecules, such as nucleic acids, lipids, proteins, and even pharmacological compounds (8).

Over the past decades, researchers have been investigating the role of EVs in breast cancer. Studies have demonstrated that EVs are extensively involved in the major pathways implicated in breast cancer development, including proliferation, migration, tumor microenvironment (TME), and drug resistance (9, 10). Moreover, multiple clinical studies have demonstrated the potential applications of EVs in the treatment and diagnosis of breast cancer (11–13). This review attempts to provide a theoretical basis for the treatment and diagnosis of breast cancer by focusing on the promotive role of EVs in breast cancer development, the potential of EVs as breast cancer biomarkers, the mechanistic action of EVs in regulating drug resistance, and the role of EVs in anti-cancer treatment.

## 2 Roles of EVs in the development of breast cancer

### 2.1 Effects of breast cancer-derived EVs on tumor growth and migration

Accumulating evidence has demonstrated a strong link between glucose homeostasis and the growth of breast cancer cells. Cao et al. have demonstrated that miR-122, an EVs cargo derived from breast cancer cells, inhibits glycolysis and ATP-dependent insulin exocytosis and disrupts the critical function of islets to maintain normal blood glucose levels. This effect further suppresses insulin secretion, augments endogenous glucose production, impairs glucose tolerance, and disrupts fasting hyperglycemia. As a result, all these effects on glucose homeostasis could promote the rapid growth of breast cancer (5, 14, 15). In addition, breast cancer cells have been found to deliberately deviate trajectories during migration with the help of EVs and hyaluronic acid. This explains the role of EVs and hyaluronic acid in guiding the migration and metastasis of tumor cells (16). A study by Eckhard et al. confirmed the role of exosomal Rab27a in breast cancer development and metastasis, which indicated that targeting Rab27a reduced the release of exosomes, thus retarding breast cancer growth (17). Wanessa et al. have demonstrated that adhesion receptors, previously thought to be expressed only at the cellular level, are additionally present on SEVs membranes and thus participate in processes such as adhesion and uptake. Specifically, αvβ3 integrins, adhesion receptors present on SEVs, secreted by triple-negative breast cancer cells (MDA -MB-231) have been reported to accelerate invasion and organ-specific metastasis of breast cancer cells. Moreover, the downregulation of αvβ3 integrin expression further reduces the expression of the pro-oncoprotein CD63 present on the SEVs membrane, thereby inhibiting breast cancer progression (18). A *in vitro* study by Bertolini et al. highlighted that SEVs released from breast cancer cells activated NF-κB pathway in breast epithelium cells under hypoxic conditions, then promoted the release of inflammatory cytokines and mitochondria-mediated cell migration, which enabled the disruption of the mammary glandular vesicle structure. Simultaneously, this hypoxic microenvironment in patients with breast cancer commonly contributed to local carcinogenic and oncogenic inflammatory changes (19). Another study has found that Caveolin-1 is upregulated in metastatic breast cancer cell-secreted EVs and fosters breast cancer invasion and metastasis through the adhesion proteins (20). Besides, Wu et al. found that the bone marrow mesenchymal stem cell (BMSC)-derived EVs could be internalized by breast cancer cells and significantly improve the proliferation ability of these breast cancer cells (21). Adipocytes are the most abundant stromal cell component in breast cancer tissues and support the survival of metastatic breast cancer cells at distant sites (22). Reportedly, exosomes from adipose tissue-derived MSCs are internalized by breast cancer cells (MCF7), which activate the Hippo pathway to facilitate the proliferation and migration of breast cancer cells (23). Genomic instability (GI) is a major driver of tumorigenesis (24, 25). Specifically, genomic changes tend to affect cellular phenotypes and are evolutionary hallmarks of most cancers (26). As illustrated in the work of Siqi et al., three miRNAs (miR-421, miR-128-1, and miR128-2) were identified from 18 GI-associated miRNAs contained in EVs from serum of breast cancer patients, which were defined as GI-derived miRNA signature (miGISig). By analyzing its correlation with patient clinicopathological data from publicly available breast cancer -associated datasets (such as TCGA) and *in vitro* functional experiments, miGISig was found to be closely associated with poor prognosis of patients, indicating its high diagnostic potential for breast cancer (27). These results suggest that EVs derived from breast cancer cells can stimulate the growth and migration of tumor cells, which in turn facilitates the malignant progression of tumors.

### 2.2 Effect of EVs on angiogenesis

Angiogenesis, the formation of new blood vessels from pre-existing blood vessels, is an important cause of tumor growth and blood-borne metastasis. This is a dynamically complex process involving multiple mechanisms, regulated by a variety of cancer related molecules (28). In healthy tissues, angiogenesis is tightly regulated by a precise balance between stimulating and inhibiting signals. When this balance is disturbed, abnormal vascular growth occurs and is a major cause of breast cancer invasion and metastasis (29). EVs are important mediators of angiogenesis through constant communication with the environment through a variety of para-secretory factors and cell-to-cell and cell-to-matrix interactions (30). Moreover, EVs released by primary tumors also promote angiogenesis and cancer progression. Aslan et al. have found that docosahexaenoic acid downregulates the expression of pro-angiogenic genes, such as *HIF1-α, TGF-β, SOX2, Snail1, Snail2*, and *VEGFR*, in breast cancer cell-derived exosomes. Furthermore, the docosahexaenoic acid inhibits tumor angiogenesis by upregulating tumor-suppressing miRNAs (miR-101, miR-199, and miR-342) and downregulating oncomiRs (miR-382 and miR-21) in exosomes (31). In addition, MSC-derived EVs repress the expression of vascular endothelial growth factor (VEGF) by miR-100/mTOR/HIF1α signal axis, ultimately retarding the formation of capillary-like tubules in endothelial cells (32). Meanwhile, MSC-derived exosomes deliver miR-16 to repress the expression of its target *VEGF*, thus curtailing angiogenesis in breast cancer (33). Sayantan et al. have demonstrated that, in primary breast cancer cell-derived SEVs, annexin II induces angiogenesis both *in vitro* and *in vivo* by upregulating the expression of the tissue fibrinogen activator tPA, which in turn promotes lung and brain metastases of breast cancer (34). Moreover, breast cancer cell-derived exosomes stimulate endothelial cell angiogenesis in the TME *via* the circHIPK3/miR-124-3p/MTDH axis (35). These data suggest that breast cancer cell-derived EVs can stimulate angiogenesis in the TME, which in turn promote breast cancer development and progression. Although many studies have demonstrated the effect of EVs on angiogenesis, based on the presented findings and clinical experiences, we can easily imply that another severe adverse effect associated with EVs administration may be an increased risk of thrombosis. Therefore, further preclinical models are needed to better evaluate the risk and benefit and more attention should also be paid to this point.

### 2.3 Tumor-derived EVs facilitate pre-metastatic niche formation

Cancer metastasis can be promoted by forming a supportive TME at secondary organ sites, called PMN, wherein the EVs secreted by tumor cells can modulate TME changes (36). Busatto et al. have suggested that EVs secreted from brain metastatic cells of breast cancer induce low-density lipoprotein aggregation, which accelerates uptake of these metastatic breast cancer cells by monocytes, and promotes the formation of a PMN in the brain (37). Reportedly, EVs secreted by the triple-negative breast cancer cell lines MDA-MB-231 and SUM159 have been found to induce lung fibroblasts to express the extracellular matrix (ECM) proteins fibronectin, tenascin-c, and periostin, thereby contributing to the formation of PMN in the brain (38). Moreover, LC3-loaded EVs released by breast cancer cells could induce lung fibroblasts to produce CCL2 by the HSP60-TLR2-MyD88-NF-κB pathway. Then, CCL2 recruits monocytes to the lung and subsequently form a pulmonary PMN characterized by monocytes, high infiltration of macrophages, immunosuppression, and enhanced vascular permeability, ultimately accelerating the formation of breast cancer lung metastasis (39). Lin28B has been considered a potent inducer of breast cancer metastasis, and neutrophil N2 conversion is a key step in the suppression of T-cell function. Specifically, Qi et al. have found that Lin28B is involved in neutrophil recruitment and N2 conversion through let-7s-carrying exosomes to alter the immune status in the TME, thereby establishing an immunosuppressive PMN and ultimately inducing lung metastasis of breast cancer (40). According to the findings of Wu et al., miR-19a and integrin-binding sialoprotein (IBSP) expression in breast cancer cells fail to alter the *in vitro* growth or migration ability of breast cancer cells. However, following encapsulation and release by exosomes, IBSP can attract osteoclasts and form an osteoclast-rich microenvironment to assist the delivery of exosomal miR-19a to osteoclasts, thus further inducing osteoclast formation. This subsequently augment PMN formation and bone metastasis in estrogen receptor (ER)-positive breast cancer (41). As previously reported, exosomes secreted by the mildly metastatic MDA-MB-231 cell line and the highly metastatic SCP28 breast cancer cell line could release miR-21 and subsequently stimulate PMN formation by upregulating programmed cell death (PD) 4 protein levels, whereby strengthening osteoclast differentiation and activation, ultimately leading to bone metastasis of breast cancer (42). From above findings, we could imply that the formation of PMN caused by tumor-derived EVs could significantly assist in organ colonization and metastasis of breast cancer, such as brain, lung and bone. As a result, patients died from tumor metastasis. In the future, we should focus on developing the novel methods to block the formation of PMN caused by tumor-derived EVs, and effectively prevent the happening of organ-specific tumor metastasis.

### 2.4 Tumor-derived EVs promote metastasis

Despite significant advances in novel anti-cancer therapies for advanced breast cancer in recent years, many patients with breast cancer remain incurable and metastasis remains the leading cause of death in patients with advanced breast cancer (43, 44). Tumor metastasis is a multistep process involving the spread of cancer cells from the primary tumor to the blood or lymphatic system, then survival in circulation, subsequent migration to target organs, and eventually distant implantation and proliferation (45, 46). These effects require tight regulation of the cellular mechanisms by which tumor cells detach from the primary tumor and transfer to the metastatic site, in which EVs also play a crucial role (47, 48). The work of Shechter et al. has exhibited that highly metastatic or chemo-resistant breast cancer cells release more EVs than breast cancer cells with low metastatic potential. Additionally, these EVs reduce cell adhesion by substantially expressing CD44, which subsequently disrupt actin filament structure and ultimately induces tumor spread, thus contributing to breast cancer metastasis (49).

Bone is the most likely site of metastasis for all molecular subtypes of breast cancer. Patients with bone metastases often have other serious complications, such as severe bone pain, fractures, severe hypercalcemia, and nerve compression syndrome, This seriously affects the life expectancy and the quality of life (50). Under normal conditions, bones undergo a dynamic balance of bone resorption and bone formation mediated by osteoclasts and osteoblasts, respectively. However, bone metastases usually show an imbalance in this process (51), and osteolytic bone metastases is the most common type (52). EVs also promote this process. EVs can introduce miR-940 into the bone to promote osteogenic differentiation of mesenchymal stem cells by targeting Rho GTase-activating protein 1 (ARHGAP1) and FAM134A. As a result, promoting osteolytic bone metastasis (53). MDA-MB-231 triple-negative breast cancer cells also secrete EVs loaded with miR-218 to block osteoblast differentiation, tip the balance toward osteolysis, and form metastatic bone niches to facilitate osteolytic bone metastasis (54). Meanwhile, ER^+^ breast cancer cells can also produce EVs loaded with miR-19a, and promote osteolytic bone metastasis by inhibiting PTEN expression and inducing NF-κB and AKT pathways (41). So, in the future, targeting EVs to inhibit the formation of osteolytic bone metastasis may be a promising research direction in the field of cancer metastasis.

Lung is another important target organ of frequent breast cancer metastasis. Pulmonary capillaries consist of endothelial cells surrounded by basal membranes and adjacent alveolar cells. Tumor cells need to adhere to this endothelial membrane and penetrate into the lung parenchyma to establish a metastatic tumor (55, 56). Tumor-derived EVs secreted by highly metastatic breast cancer cells could load miR-122 to increase nutrient availability in lung metastatic cancer cells by down-regulating the glycolytic enzyme pyruvate kinase in lung fibroblasts, as a result, facilitating the formation and development of breast cancer lung metastasis (57). EVs also mediate miRNA transfer from cancer cells to other cells for tumor microenvironmental remodeling and promote the formation of pre-metastatic niches in lung. miR-138-5p and miR-183-5p loaded by EVs and secreted by cancer cells can regulate the activity of tumor-associated macrophages (TAM) by targeting KDM6B and PPP2CA respectively, and to promote the formation of breast cancer lung metastasis (58, 59). Besides miRNA, other non-coding RNA loaded in EVs also contribute to the formation of breast cancer lung metastasis. Abnormal expression of lncRNA in EVs promoted the formation of lung metastasis microenvironment (60, 61). Moreover, Exosome circPSMA1 could up-regulate Akt1 expression and affect the expression of downstream genes such as β-catenin and cyclin D1. circPSMA1/miR637/Akt1/β-catenin (cyclin D1) signal axis promotes the lung metastasis of triple-negative breast cancer (62). We believe that blocking the formation of lung-specific breast cancer metastasis could also affectively improve the survival outcomes of patients with breast cancer.

Brain metastasis occur in about 10%–30% of patients with breast cancer (63). Since most chemotherapy drugs can’t cross the blood-brain barrier (BBB), brain metastasis is refractory to conventional chemotherapy and exhibit extremely poor survival outcomes (64, 65). Therefore, preventing breast cancer cells from infiltrating the BBB is an important step in preventing and treating the brain metastases of breast cancer. Specifically, cytoskeleton-associated proteins, which are involved in endothelial cell adhesion and migration at the BBB, are important targets in preventing breast cancer metastases into the brain (66). The tight association between the increased expression of the cytoskeleton-associated protein tubulin tyrosine ligase like 4 (TTLL4) and brain metastasis of breast cancer was proposed by Arnold et al. They reported that TTLL4-dependent β-tubulin glutamylation increased the secretion of EVs synthesized by multivesicular bodies. These EVs increased the ability of breast cancer cells to adhere to BBB endothelial cells as well as the permeability of these endothelial cells, ultimately promoting breast cancer brain metastasis (67). Furthermore, EVs originated from vascular endothelial cells were found to induce the adhesion of triple-negative breast cancer cells to endothelial cells, and this process could be enhanced by cirGal-3. Further mechanistic exploration revealed that the enhanced glycolysis of endothelial cells induced by cirGal-3 could increase ICAM-1 expression; and EVs-dependent delivery of cirGal-3 to triple-negative breast cancer cells (MDA-MB-231) increased adhesion between the two cell types, ultimately leading to brain metastases of breast cancer (68). Moreover, Sirkisoon et al. have revealed that miR-1290-containing EVs derived from breast cancer activate astrocytes in brain TME *via* the FOXA2-CNTF axis. This subsequently augmented intracranial colonization and growth of breast cancer cells, leading to the progression of brain metastasis (69).

However, how do breast cancer cells acquire the ability to release specific EVs cargo to maintain the plasticity of breast cancer and the rapid spread of tumor metastasis? Further research should be performed to answer this complex question and more strategies should be proposed to block the process.

## 3 Roles of EVs in TME

Tumor development is not only influenced by intrinsic characteristics but also regulated by signals from the TME. In addition, a constant exchange of information between cancer cells and the TME is crucial to sustain tumor growth, angiogenesis, and spread. Tumor stromal cells, including fibroblasts, immune inflammatory cells, vascular endothelial cells, and others play a crucial role in tumor response to therapy and influence tumor cell proliferation, invasion, and metastasis. Interactions between cellular and non-cellular components of the TME underly pro-tumorigenic signaling, which augments tumor growth and metastasis (70–72). Specifically, EVs play an important role in these regulatory processes of tumor progression by mediating signaling from malignant tumor cells to non-tumor cells and the ECM (6, 73–75).

### 3.1 Effects of EVs on fibroblasts

Cancer-associated fibroblasts (CAFs) are a group of activated fibroblasts in the TME with significant heterogeneity and plasticity. Particularly, CAFs are involved in tumor progression-related regulatory processes, such as the regulation of tumorigenesis, metastasis, and therapeutic resistance (76). Fibroblasts can engulf EVs secreted by breast cancer cells, in turn, fibroblasts are activated to become CAFs and stimulate the carcinogenic phenotype of breast cancer (77). Moreover, EVs released from CAFs can be engulfed by breast cancer cells to subsequently drive their migration, invasion, proliferation, epithelial-mesenchymal transition (EMT), chemoresistance, and ECM remodeling (78). Prior evidence by Baroni et al. suggests that triple-negative breast cancer cells stimulate the conversion of fibroblasts to CAFs through EVs cargo miR-9. Moreover, these newly transformed CAFs can secrete miR-9 to augment breast cancer invasion and metastasis by increasing E-cadherin expression (79). Specifically, Vu et al. found that the transfer of miR-125b-containing EVs from breast cancer cells to normal fibroblasts significantly increased the expression of the CAFs activation markers Acta2, MMP2, and MMP3, thereby promoting the conversion of CAFs phenotype (80). Concurring results by Yang et al. demonstrated that EVs released from breast cancer cells (MDA-MB-231 and MCF-7), which transferred miR-146a to normal fibroblasts, then activated the Wnt/β-catenin pathway and subsequently induced the malignant phenotypes of CAFs (81). Nevertheless, Tao et al. highlighted that EVs released from CAFs can impede the growth and metastasis of breast cancer cells by inhibiting EMT and proliferation *via* upregulating miR-1-3p expression (82). We can easily imply from these reported results that both breast cancer cells and CAFs can secrete EVs to regulate breast cancer progression, but the specific oncogenic or tumor-suppressive effect depends on the different effector proteins. However, more depth mechanisms underlying the effect of EVs on the behaviors of CAFs require further investigation.

### 3.2 Effects of tumor-derived EVs on immune cells

Chronic inflammation is an important driver of tumorigenesis and progression (83, 84). Neutrophils are the most abundant leukocytes in the immune system and the first to migrate to sites of inflammation (85). These cancer-associated neutrophils exhibit both anti-tumor (N1) and pro-tumor (N2) phenotypes, with N1 neutrophils having a stronger cytotoxic effect on tumor cells and N2 neutrophils supporting tumor cell growth, invasion, and metastasis (86). As identified by Amorim et al., EVs released from breast cancer cells (MDA-MB-231) induced the N2-phenotype transformation of neutrophils. These MDA-MB-231-secreted EVs enhanced the production of neutrophil extracellular traps, interleukin (IL)-8, and VEGF from N2 neutrophils, as well as increased the expression of Arg-1, MMP9, and the N2 neutrophil marker CD184, which subsequently promoted breast cancer cell invasion (87). Reportedly, PD-L1 is overexpressed in breast cancer cells and is considered a key tumor immunosuppressor (88, 89). Specifically, PD-L1 maintains the immunosuppressive microenvironment by interacting with PD-1 on lymphocytes (90, 91). CAF-derived exosomes overexpress miR-92 *via* YAP1, and this increases PD-L1 expression to impair the function of tumor-infiltrated immune cells to drive breast cancer progression (88, 92). As previously documented by Xie et al, breast cancer cell-derived EVs transfer active TGF-β type II receptors (TβRII) to activate TGF-β signaling in target cells. The uptake of TβRII-containing EVs by breast cancer cells with low metastatic potential triggers EMT, which enhances the stemness and metastatic ability of breast cancer cells. Meanwhile, EVs-dependent delivery of TβRII to CD8^+^ T cells also induces the activation of SMAD3 and TCF1, further causes CD8^+^ T cell depletion and subsequent failure of tumor immunotherapy (93). Besides, Njock et al. identified that three miRNAs (miR-142-5p, miR-183-5p, and miR-222-3p) were released from endothelial cells in the TME and were transferred *via* EVs to macrophages. In a mouse model of breast cancer, EVs enriched with these miRNAs contributed to the differentiation of macrophages toward the immunosuppressive M2 phenotype, which augmented breast cancer growth (94).

Myeloid-derived suppressor cells (MDSCs) are closely associated with breast cancer progression (95, 96). Jiang et al. highlights that miR-9 and miR-181a shuttled by EVs from breast cancer cells could activate the JAK/STAT signaling pathway by targeting SOCS3 and PIAS3. This fosters the expansion of early MDSCs and compromises T-cell proliferation and promotes T-cell apoptosis, which subsequently accelerates the growth of breast cancer cells both *in vivo* and *in vitro* (97). Biswas et al. further indicated that exosomes secreted by MSCs can harbor high levels of TGF-β, which subsequently induced the malignant progression of breast cancer by facilitating the differentiation of immature MDSCs into M2 macrophages with stronger immunosuppressive activity. Exosomes derived from MSCs, but not from breast cancer cells, contain significant amounts of TGF-β, C1q, and semaphorins. Moreover, the induced PD-L1 overexpression in immature myelomonocyte precursors and CD206^+^ macrophages, and the fostered differentiation of MHC class II macrophages enhance Arg-1 activity and IL-10 secretion to promote myeloid tolerance activity. This ultimately leads to the formation of immunosuppressive M2 macrophages and results in the rapid growth of breast cancer cells (98). The interactions between EVs cargoes and immune cells significantly contribute to the rapid progression of breast cancer, and also facilitate to the formation of immunosuppressive tumor microenvironment.

### 3.3 Effects of tumor-derived EVs on cell dormancy

Tumor cells undergo EMT before the occurrence of tumor metastasis. During EMT, tumor cells lose epithelial features such as cell adhesion and acquire mesenchymal features, which allow them to migrate and metastasis to occur (99–101). When tumor cells reach the metastatic site, they then undergo the reverse process called mesenchymal-epithelial transformation, which allows these cells to colonize their metastatic site (102–104). However, in the early stages of metastatic spread, disseminated tumor cells may remain dormant in the G0/G1 phase to accumulate the capacity for bone marrow metastasis; this process is also observed in metastatic breast cancer cells (105). Casson et al. revealed that bone marrow MSC (BMSC)-derived EVs increase the adhesion capacity of ER^+^ breast cancer cells (MCF7) to a dormant epithelial cell phenotype. This facilitates their colonization in the bone marrow in a circulating quiescent state, ultimately leading to bone metastases of breast cancer and resistance to chemotherapeutic agents (106). Further evidence has suggested that breast cancer cells augment BMSCs to release EVs containing miR-222 and miR-223. These EVs, in turn, stimulate the dormancy of disseminated breast cancer cells, causing resistance to chemotherapeutic agents. Based on this, Bliss et al. found that, in a mouse model, targeting dormant breast cancer cells with miR-222 and miR-223 antagonists (delivered by EVs secreted by BMSC) significantly improved the susceptibility of breast cancer cells to carboplatin and also extended the survival time of mice (107). Therefore, we believe that targeting dormant breast cancer cells by EVs may be an effective strategy to improve the response to routine therapy and even prevent breast cancer recurrence and metastasis.

## 4 EVs and biomarkers of breast cancer

Connexin-46 (Cx46) is highly expressed in EVs released from breast cancer cells and can enforce the interactions between EVs and receptor cells, thereby enhancing the migratory and invasive ability of breast cancer cells. This highlights the potential of EVs-Cx46 as a malignancy marker of breast cancer and as a potential target for breast cancer therapy (108). Inflammatory breast cancer is a rare and highly aggressive malignant tumor of the breast that is easily misdiagnosed as mastitis, which results in delayed treatment (109). The EVs extracted from the plasma of patients with inflammatory breast cancer were found to have three miRNAs (miR-181b-5p, miR-222-3p, and let-7a-5p). The receiver operating characteristic curve analysis revealed an area under the curve (AUC) of > 0.9, which indicated that the three miRNAs could be extremely promising diagnostic biomarkers for patients with inflammatory breast cancer (110).

Axillary lymph node (ALN) metastasis is one of the most important prognostic factors in early-stage breast cancer, and sentinel lymph node biopsy (SLNB) is the main method to assess ALN status (111). Nevertheless, the false-negative rate of SLNB and the presence of associated complications such as postoperative lymphedema limit the use of SLNB (112, 113). Therefore, breast cancer patients without ALN metastasis would greatly benefit from avoiding SLNB and instead accurately assessing ALN status preoperatively. A study by Wang et al. has revealed that miR-363-5p was expressed at lower levels in plasma exosomes of patients with ALN-positive breast cancer than in patients with ALN-negative breast cancer. Moreover, miR-363-5p in plasma exosomes has a high diagnostic value in distinguishing ALN-positive patients from ALN-negative patients. In addition, high expression of miR-363-5p in plasma exosomes appreciably prolonged the survival of patients with breast cancer. These results confirmed that miR-363-5p-containing plasma exosomes exert oncogenic effects in breast cancer and have predictive value for non-invasive LN staging and breast cancer prognosis, indicating its potential as a diagnostic marker for breast cancer (114).

Liquid biopsy is an ideal method for studying the risk of recurrence and early detection of systemic dissemination in patients with tumors (115, 116). Multiple miRNAs contained in breast cancer cell-secreted exosomes can be used as novel breast cancer biomarkers (117, 118). Based on this, Baldasici et al. postulated a new method for the early diagnosis of breast cancer using non-invasive detection and isolation of miRNAs in tumor-derived exosomes. They identified exosomal miRNAs for use as biomarkers of breast cancer metastasis, including miRNAs associated with lymph node metastasis (miR-363-5p, miR-370-3p, miR-222, miR-148a, miR-3662, miR-146a, miR-129, and miR-188-5p), with bone metastasis (miR-21 and miR-218-5p), with brain metastases (miR-576-3p, miR-130a-3p, and miR-181c), and with distant metastases without organ specificity (miR-105, miR-200c, miR-141, and miR-7641) (119).

Of note, Buentzel et al. identified eight metabolites with a significant discriminatory power using mass spectrometric analysis of microvesicles in the blood of patients with breast cancer and healthy controls. Among these, high concentrations of lysoPCaC26:0 and PCaaC38:5 were strongly associated with poor prognosis in patients with breast cancer. In addition, an analysis of metabolites specific to breast cancer subtypes revealed 24 metabolites with significantly different expression in plasma micro-vesicles of patients with luminal A and luminal B type breast cancer, indicating the role of these metabolites in differentiating the molecular subtypes of breast cancer. Thus, metabolites in the plasma microvesicles of these patients might be promising biomarkers for breast cancer and are closely associated with breast cancer progression (120). In another study, Cai et al. analyzed mRNAs carried by EVs in patients with breast cancer and those with benign breast lesions, using a second-generation sequencing method. They identified eight mRNAs with high diagnostic values for breast cancer, namely *HLA-DRB1*, *HAVCR1*, *ENPEP*, *TIMP1*, *CD36*, *MARCKS*, *DAB2*, and *CXCL14*. The combined AUC of these mRNAs for breast cancer diagnosis was 0.718. Validation in other cohorts confirmed the high diagnostic potential of these mRNAs for breast cancer (combined AUC of validation cohort: 0.737). The study by Chen et al. noted significantly higher phosphorylation of proteins such as RALGAPA2, PKG1, and TJP2 in plasma EVs of patients vs. healthy controls. In addition, these phosphorylated proteins were considered to be viable markers for breast cancer and could assist in tumor screening and surveillance (9, 121). These results implied that certain cargoes of EVs can be used as biomarkers for breast cancer, with high clinical value in the diagnosis, treatment, and even survival outcomes. The main EVs cargoes which can be applied as biomarkers in breast cancer are also summarized and presented in **Table 1**.

**Table 1:** The EVs cargoes which can be applied as biomarkers in breast cancer mentioned in this review.

EVs cargoes	Function
Connexin-46 (Cx46)	Breast cancer migration and invasion
miR-181b-5p, miR-222-3p, let-7a-5p	Diagnosis of inflammatory breast cancer
miR-363-5p	Distinguish between lymph node positive and lymph node negative breast cancer patients
miR-363-5p, miR-370-3p, miR-222, miR-148a, miR-3662, miR-146a, miR-129, miR-188-5p	Associated with lymph node metastasis
miR-21, miR-218-5p	Breast cancer with bone metastasis
miR-576-3p, miR-130a-3p, miR-181c	Breast cancer with brain metastasis
miR-105, miR-200c, miR-141, miR-7641	Associated with distant metastasis of breast cancer, but organ-specific
lysoPCaC26:0 and PCaaC38:5	Molecular subtypes of breast cancer prognosis
HLA-DRB1, HAVCR1, ENPEP, TIMP1, CD36, MARCKS, DAB2, CXCL14	Breast cancer diagnosis
The Phosphorylation of RALGAPA2, PKG1 and TJP2	Screening and monitoring of breast cancer

## 5 Roles of EVs in drug resistance of breast cancer

Resistance to treatment is a significant clinical challenge in the treatment of patients with breast cancer as it is often associated with treatment failure (122, 123). Moreover, bidirectional regulation between breast cancer cells and the TME has been established to be an important factor in breast cancer treatment resistance, with crosstalk between EVs and the immune microenvironment playing an important role (124–126). Abad et al. have delineated that EVs secreted by chemo-resistant triple-negative breast cancer cells could transfer mitochondria to chemo-sensitive cancer cells, thereby increasing their chemoresistance. In addition, EVs carrying mitochondria from drug-resistant triple-negative breast cancer cells promoted chemoresistance by increasing the level of mtDNA with mutations in the *mtND4* gene, which was responsible for tumorigenesis. Furthermore, the exosome releasing inhibitor GW4869 was found to inhibit mitochondrial transport and subsequently reduce chemotherapy resistance in triple-negative breast cancer cells (127).

As over 70% of breast cancer are hormone receptor-positive (128), and endocrine therapy remains the most effective treatments for ER^+^ breast cancer (129, 130). Tamoxifen is the gold standard of endocrine therapy in breast cancer; however, tamoxifen resistance has been a major concern for clinicians (131, 132). Recent studies have demonstrated that EVs play a crucial role in conferring resistance to endocrine therapy. Moreover, miRNAs can be expressed as a “rich cocktail” and delivered to cells with exosomes. Among them, miRNAs involved in the negative regulation of ERs, such as miR-181a-2, when delivered to target cells, could further induce the rearrangement or interdiction of hormonal signaling or the activation of the PI3K/AKT signaling pathway, ultimately reducing the sensitivity of ER^+^ breast cancer cells to endocrine therapy (133). In a study, Wang et al. reported the upregulation of transient receptor potential canonical 5 (TRPC5) expression as an important factor for chemoresistance in breast cancer cells. Specifically, chemo-resistant breast cancer cells can release EVs containing TRPC5, which is then delivered to chemo-sensitive cells, subsequently leading to acquired chemoresistance. Further studies have found that TRPC5 levels in plasma EVs are significantly correlated with TRPC5 levels in breast cancer tissues and chemotherapy response. Moreover, elevated TRPC5 levels in plasma EVs after chemotherapy have been reported to strongly predict acquired chemoresistance in patients with breast cancer. Based on these findings, TRPC5 levels in plasma EVs after chemotherapy may serve as a valid predictive marker for acquired chemoresistance (134). A study by Wang et al. has noted an increased level of long non-coding RNA H19 in EVs derived from adriamycin-resistant breast cancer cells compared to control cells. Moreover, repression of H19 levels in EVs potentiates the sensitivity of breast cancer cells to adriamycin. This implied that H19 carried by EVs can influence adriamycin resistance in breast cancer cells (135). The use of aromatase inhibitors (AIs) is the standard endocrine therapy for postmenopausal patients with ER^+^ breast cancer (136, 137); however, its efficacy is limited owing to the development of drug resistance. A study by Augimeri et al. demonstrated that AI-resistant MCF7 cells released more EVs to participate in AI resistance than control ER^+^ MCF7 cells. The proteomic analysis further confirmed that Rab GTPases, an important vesicular transport regulatory protein in tumors, was expressed at significantly high levels in EVs from AI-resistant MCF7 cells, which may be a major factor causing the increased secretion of EVs as well as AI resistance (138).

**Figure 1:**
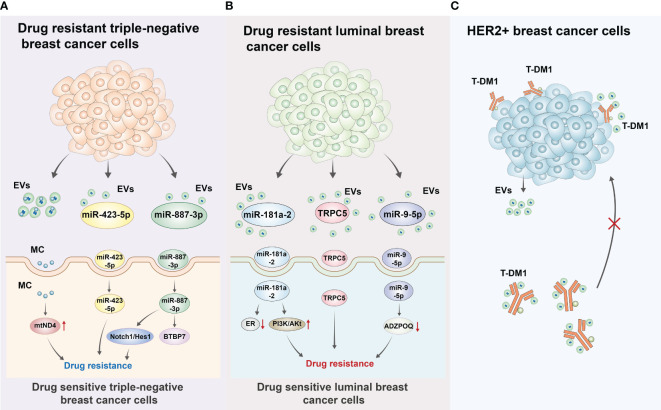
Roles of EVs in resistance to the treatment of breast cancer **(A)** Drug resistant triple-negative breast cancer cells can secrete EVs including mitochondrion (MC), miRNA (miR-423-5p, miR-887-3p) to drug sensitive triple-negative breast cancer cells. For MC, it can induce gene mutation of *mtND4* to cause drug resistance. For miR-423-5p, miR-423-5p-enriched EVs could convert cisplatin-sensitive breast cancer cells into cisplatin-resistant breast cancer cells by activating proliferation, metastasis, and anti-apoptosis-related signaling pathways. For miR-887-3p, it could target BTBP7 and activate Notch/Hes1 pathway to induce drug resistance of triple-negative breast cancer cells. **(B)** Drug resistant luminal breast cancer cells can also secrete EVs including miR-181a-2, TRPC5, miR-9-5p to drug sensitive luminal breast cancer cells. For miR-181a-2, it can contribute to the interdiction of ER signal and activation of PI3K/AKT to induce drug resistance. TRPC5 could convert cisplatin-sensitive breast cancer cells into cisplatin-resistant breast cancer cells. For miR-9-5p, it can lead to tamoxifen treatment resistance by downregulating the expression of its target gene *ADIPOQ*. **(C)** This diminished efficacy of antibody-drug conjugate therapy may be ascribed to the binding of T-DM1 to HER2^+^ breast cancer cell-secreted exosomes, which eventually resulted in treatment resistance.

Trastuzumab emtansine (T-DM1) is an antibody-drug conjugate designed to specifically deliver the cytotoxic drug DM1 (a derivative of maytansine) to HER2^+^ breast cancer cells (139–141). Despite the significant efficacy of T-DM1 to target HER2^+^ breast cancer cells, most patients with HER2^+^ breast cancer treated with T-DM1 experience local recurrence or distant metastases after drug discontinuation. In addition, a proportion of patients with HER2^+^ breast cancer developed resistance to T-DM1 (141–143). Oya et al. elaborated that this diminished efficacy of antibody-drug conjugate therapy may be ascribed to the binding of T-DM1 to HER2^+^ breast cancer cell-secreted exosomes, which eventually resulted in treatment resistance (144). In addition, other studies have found that MSC-derived exosomal miR-342-3p inhibited metastasis and chemoresistance of breast cancer cells by targeting *ID4* (145). In line with this, Liu et al. observed robust expression of miR-9-5p in EVs derived from ER^+^ breast cancer cells (MCF7), which could lead to tamoxifen treatment resistance by downregulating the expression of its target *ADIPOQ* (146). Moreover, prior evidence has clarified that EVs derived from cisplatin-resistant MDA-MB-231 cells have high expression of miR-423-5p. Furthermore, miR-423-5p-enriched EVs could convert cisplatin-sensitive breast cancer cells into cisplatin-resistant breast cancer cells by activating proliferation, metastasis, and anti-apoptosis-related signaling pathways (147). Available evidence suggests that the MDA-MB-231 cell-derived EVs can stimulate chemoresistance by shuttling miR-887-3p to target *BTBD7* and thus activate the Notch1/Hes1 signaling pathway (148). As illustrated by Yang et al., adriamycin and paclitaxel can activate breast cancer cells to secrete miR-378a-3p- and miR-378d-enriched EVs. Then, these EVs can be internalized by breast cancer cells and subsequently activate the Wnt and Notch signaling pathways by targeting *DKK3* and *NUMB via* miR-378a-3p and miR-378d. This ultimately led to increased cell stemness and the acquisition of a drug-resistant phenotype (149). These results predicted that EVs cargoes also participated in the formation of drug resistance of breast cancer. Therefore, targeting these EVs cargoes may increase the therapeutic effect of anti-tumor drugs, including chemotherapy, endocrine therapy, targeted therapy and even immune therapy. The roles of EVs in resistance to the treatment of breast cancer were also summarized and presented in **Figure 1**.

## 6 Roles of EVs in breast cancer clinical treatment

Finally, this study examines the important roles that EVs played in breast cancer clinical treatment. Targeting the conversion of the M1 to M2 phenotype of TAMs is an effective breast cancer treatment (150–152). Of note, the M1 phenotype of TAMs exerts pro-cancer effects, whereas the M2 phenotype exerts cancer-suppressive effects. In this regard, Zhao et al. proposed a new system to load docetaxel into M1 TAM-derived exosomes and found that the conversion of the M1 phenotype to the M2 phenotype, thus significantly improving the anti-cancer effect with minimal side effects (153). In addition, another EVs-based drug delivery system demonstrated a good synergistic anti-tumor effect, which increased tumor-killing efficiency by 15% by promoting the drug’s tumor-targeting capabilities (154). Various microbiota has been shown to play a crucial role in cancer progression as well as in treatment (155, 156). A study by An et al. expounded that the combination of *Klebsiella pneumoniae*-derived EVs and tamoxifen considerably enhanced the therapeutic effect of tamoxifen on ER^+^ MCF7 cells by downregulating cyclin E2 and p-ERK expression (157). In line with this, evidence has suggested the role of microbiome in mediating breast cancer. Specifically, the microbiome with the ability to regulate estrogen metabolism is known as the estrogenome (158). It modulates estrogen levels in the body and is strongly associated with the development of various cancers, including breast cancer. In their study, An et al. compared the EVs profiles of blood microorganisms sampled from both patients with breast cancer and healthy controls and found that *Staphylococcus aureus* (*S. aureus)*-derived EVs could influence the efficacy of tamoxifen by modulating ERK- and AKT-related signaling pathways. Moreover, the combination of *S. aureus*-derived EVs and tamoxifen synergistically impeded the growth of ER^+^ breast cancer cells (159).

In addition, Chang et al. have revealed that endocytosis of EVs secreted from Wharton’s Jelly mesenchymal stem cells (WJ-MSCs) into triple-negative breast cancer cells significantly reduced the proliferation potential, stem cell characteristics, tumor formation capacity in nude mice, and metastatic capacity under hypoxic conditions of triple-negative breast cancer cells. This indicated that targeting triple-negative breast cancer with MSC-derived EVs has potential therapeutic effects. Further in-depth mechanistic analysis revealed that WJ-MSC-secreted EVs attenuated the tumorigenic ability of triple-negative breast cancer cells and prevented the formation of immunosuppression in the TME. This was facilitated by transferring miR-125b to triple-negative breast cancer cells and inhibiting HIF1α signaling pathway-related protein expression, which ultimately induced therapeutic effects (160). Using a tissue engineering approach, Gong et al. developed EVs containing therapeutic doses of adriamycin and cholesterol-modified miR-159. Their study revealed that using these EVs to target triple-negative breast cancer cells resulted in good anti-tumor effects (161). Owing to the pharmacologically important characteristics of exosomes, nanoparticles based on cell-derived exosomes, developed using tissue engineering, have considerably contributed to the treatment of tumors (162–164). Shi et al. developed the synthetic multivalent antibodies retargeted exosome platform using tissue engineering. Briefly, they modified exosomes targeting human-derived CD3 and HER2 proteins to attack HER2-expressing breast cancer cells by recruiting CD3^+^-expressing cytotoxic T cells. This ensured efficient and targeted treatment against HER2^+^ breast cancer (165). Thus, we believe that, based on the biological properties of EVs, a combination of research methods related to tissue engineering such as the application of nanomaterials and tumor cell biology helps in developing more effective anti-tumor treatments, consequently providing new alternatives for tumor treatment in clinical settings.

## 7 Conclusions and perspectives

EVs play an important role in intercellular signaling, regulating almost all tumor biological properties. In addition, they significantly affect the interaction between tumor cells and TME. Meanwhile, as a novel anti-cancer therapeutic tool, EVs are receiving considerable attention for application in clinical settings. Specifically, using tissue engineering, EVs have been found to have broad clinical application prospects. These advantages of EVs are primarily attributed to their presence at various locations in tumor cells and TME, their unique composition, and their high efficacy in targeting tumor cells. However, the extraction and purification of EVs are still challenging, and their utility as biomarkers and therapeutic targets requires further exploration. In short, this update review mainly focuses on the role of EVs in the malignant progression of breast cancer, the potential of EVs as biomarkers of breast cancer, the mechanism of EVs in regulating drug resistance of breast cancer treatment, and the prospects of EVs in breast cancer treatment. We hope that this update review could provide a theoretical basis for the treatment and diagnosis of breast cancer in the real clinical practice.

## Author contributions

XDZ and JL designed this research. XYZ, CZW, JHY, JWB, FLA, YW and XDZ retrieved relevant literature. JL and XDZ wrote this paper. All authors contributed to the article and approved the submitted version.
